# Genome-wide identification of copy number variation and association with fat deposition in thin and fat-tailed sheep breeds

**DOI:** 10.1038/s41598-022-12778-1

**Published:** 2022-05-25

**Authors:** Shadan Taghizadeh, Mohsen Gholizadeh, Ghodrat rahimi-Mianji, Mohammad Hossein Moradi, Roy Costilla, Stephen Moore, Rosalia Di Gerlando

**Affiliations:** 1 Department of Animal Science, Faculty of Animal Science and Fisheries, Sari Agricultural Sciences and Natural Resources University, P.O. Box - 578, Sari, Iran; 2 Department of Animal Science, Faculty of Agriculture and Natural Resources, Arak University, Arak, Iran; 3 Ruakura Research Centre, AgResearch, Hamilton, New Zealand; 4 Queensland Alliance for Agriculture and Food Innovation, University of Queensland, Brisbane, Australia; 5 Dipartimento Di Scienze Agrarie, Alimentari E Forestali, Università Degli Studi Di Palermo, Palermo, Italy

**Keywords:** Agricultural genetics, Animal breeding, Genetic association study, Genetic markers, Genomics

## Abstract

Copy number variants (CNVs) are a type of genetic polymorphism which contribute to phenotypic variation in several species, including livestock. In this study, we used genomic data of 192 animals from 3 Iranian sheep breeds including 96 Baluchi sheep and 47 Lori-Bakhtiari sheep as fat-tailed breeds and 47 Zel sheep as thin-tailed sheep breed genotyped with Illumina OvineSNP50K Beadchip arrays. Also, for association test, 70 samples of Valle del Belice sheep were added to the association test as thin-tailed sheep breed. PennCNV and CNVRuler software were, respectively, used to study the copy number variation and genomic association analyses. We detected 573 and 242 CNVs in the fat and thin tailed breeds, respectively. In terms of CNV regions (CNVRs), these represented 328 and 187 CNVRs that were within or overlapping with 790 known Ovine genes. The CNVRs covered approximately 73.85 Mb of the sheep genome with average length 146.88 kb, and corresponded to 2.6% of the autosomal genome sequence. Five CNVRs were randomly chosen for validation, of which 4 were experimentally confirmed using Real time qPCR. Functional enrichment analysis showed that genes harbouring CNVs in thin-tailed sheep were involved in the adaptive immune response, regulation of reactive oxygen species biosynthetic process and response to starvation. In fat-tailed breeds these genes were involved in cellular protein modification process, regulation of heart rate, intestinal absorption, olfactory receptor activity and ATP binding. Association test identified one copy gained CNVR on chromosomes 6 harbouring two protein-coding genes *HGFAC* and *LRPAP1.* Our findings provide information about genomic structural changes and their association to the interested traits including fat deposition and environmental compatibility in sheep.

## Introduction

**Figure 1:**
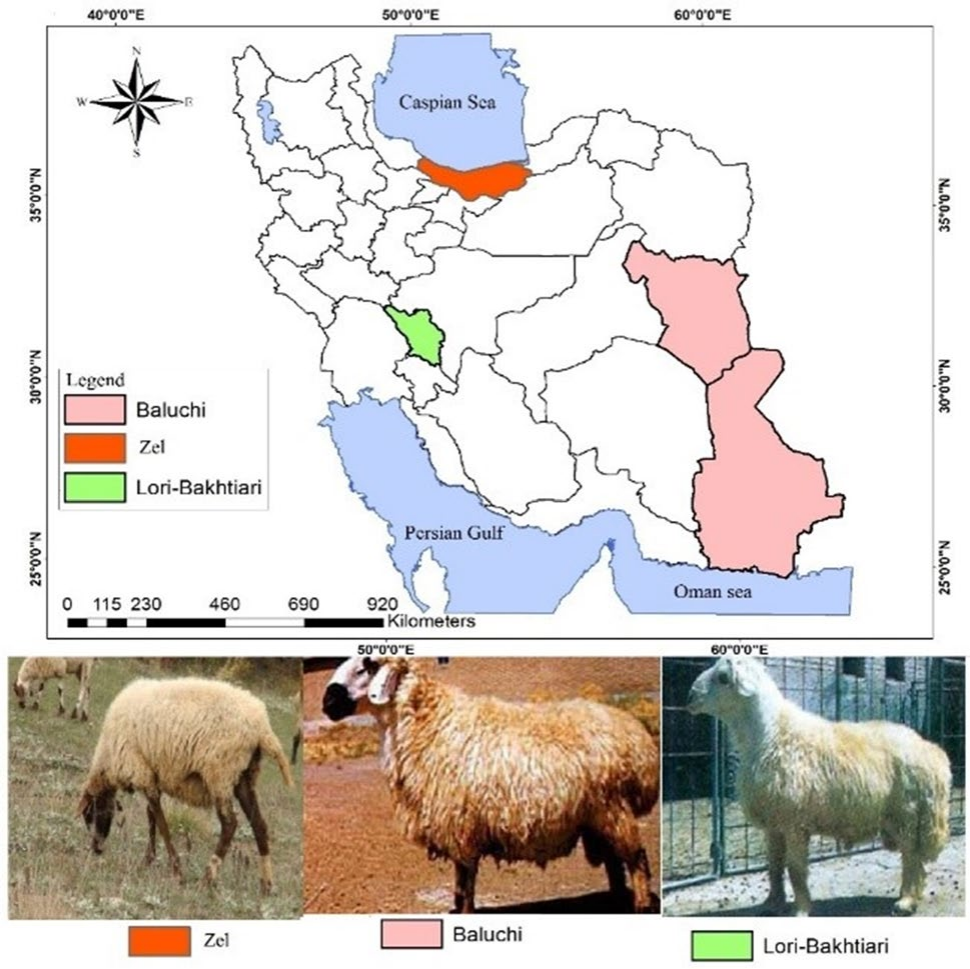
Traditional geographic distributions of the three Iranian sheep breeds.

Sheep breeding has an important role in meat production in Iran. There are 28 distinct sheep breeds in Iran distributed over different environments from dry and warm climate to the mountainous cold areas^1,2^. These breeds are characterized by a wide range of phenotypic variation especially in the case of tail shape. The vast majority of Iranian sheep are fat-tailed^3^ while Zel is known as the only thin-tailed breed reared in the north part of Iran near to Caspian Sea (Fig. 1). The fat tail characteristic of sheep has a role in the survival and adaptation mechanisms in harsh environments. It is aimed for depositing nutrients when food supply is abundant and represents a valuable metabolic energy during periods of climate changes like drought and highly cold periods and food insufficiency^4,5^. In addition, the fat in the tails can be consumed by humans as a source of energy during periods of drought and famine^6,7^.

Since a large portion of the fat deposited in the carcass of sheep is in the tail and represents more than 20% of the carcass weight, the amount of feed requirement to store fat in the tail and therefore, the cost of feeding, could be considerable^8,9^. With the development of modern livestock breeding systems, most of the benefits of a fat tail have lost their significance and accordingly reduction of fat deposition in tail is one of the main interests of the producers^7^. Besides, the consumers in many cases prefer the low-fat meat and therefore the carcass adiposity especially in the case of the fat tailed, is no longer desirable to customers and lessens the value of the meat^10^. Removing or reducing the amount of the fat in the tails of local sheep can be a breeding objective for the sheep industry. This can be approached by docking the fat-tail^11^, slaughtering lambs at an early age, or crossing the fat-tailed breeds with tailed breeds^10^.

The phenotypic diversity and genetic structure among sheep breeds appeared due to environmental adaptation and artificial selection for economically important traits such as meat, milk and wool^12^. Sheep adaptation to different environmental conditions may have resulted in variation in genes and genomic structure^13^. A better understanding of the genomic structure underlying fat deposition is very important for controlling the fat deposition in carcass through sheep breeding. With the advent of genome-wide SNP detection technology, it is now possible to identify genomic regions associated with a variety of economically important traits and subsequently to use this information in genetic improvement of livestock. Structural genetic variants represent a category of genomic changes of DNA that typically extend more than a thousand bases^14,15^. Copy number variations (CNVs) are the most prevalent type of structural genetic variation as DNA segments that are presented at a different copy number when compared to a reference genome^15^. They are observed with variable length varying from 1 kb to several Mb where duplication or deletion events can be detected^16^. CNVs play main role in genetic and phenotypic variation^17,18^, gene expression and adaptation by disrupting encoding sequences, gene structure changes and the appearance of the recessive alleles^16,19–21^. There are several experimental approaches of CNV detection including array-based comparative genomic hybridization arrays (aCGH)^22^, SNP genotyping panels^23^; and next-generation sequencing (NGS)^24^. High-throughput genotyping arrays are the most commonly implemented mainly because of their benefits of their appropriate signal-to-noise ratios, measuring total signal intensity and allelic intensity ratio altogether, which makes the explanation of results easier^25,26^. Several genomic studies have been performed to identify functional genes associated with fat deposition in sheep. Using Genomic scan of selective sweeps, three novel regions on chromosomes 5, 7 and X have been reported to be associated with fat deposition in sheep^7^. Zhang et al. using high density SNP markers identified 13 candidate genes including SMURF2, FBF1, DTNBP1, SETD7 and RBM11 associated with fat metabolism in sheep^27^. Fei et al. using miRNA seqencing identified differentially expression miRNAs in short-fat-tailed short-thin-tailed sheeps among which 17 miRNAs were related with lipid metabolism^28^.

Salehian-Dehkordi et al. using the genome-wide SNPs detected eight CNVRs associated with fat deposition in the tails in the Large-tailed and Small-tailed Han sheep horboting PPP1R11 and GABBR1 genes to be involved in fat deposition in the tails^29^. Zhu et al. using high-density SNP arrays detected genome-wide CNVs in Chinese indigenous sheep with different types of tails and reported CNVRs including genes associated with fat deposition^30^.

Yuan et al. via selection signature analysis detected 6.24 Mb of overlapped regions and 43 genes that may related to fat tail process in Chinese indigenous sheep^31^. Bakhtiarizadeh et al. implemented an expressed sequence tag (EST) study and reported candidate genes associated with tail type development and reported that the *FABP4* gene expression in the fat-tail is a main cause of fat formation^32^.

Moreover, many studies have reported the contribution of the CNVs in many traits in sheep^13,30,33–36^. It is known that the agouti duplication, affects the *ASIP* locus for white and grey coat phenotypes in sheep^33,37^. Also, CNV in the *KIT* gene causes a white coat color in pigs^38^, while the phenotype of the pea comb in chickens is affected by CNV in intron 1 of *SOX5*^39^. In addition, genome scan of CNVs in Chinese sheep identified the candidate genes related to fat deposition^40^. Despite the importance of CNVs, there are no reports on CNV structure in Iranian indigenous sheep and the mechanism responsible for differences in fat deposition between the fat-tailed and thin-tailed breeds is not yet clear. The aim of this study was to identify the characteristic of CNVs in three Iranian sheep breeds with different types of tails (Baluchi and Lori-Bakhtiari as fat-tailed breeds and Zel as the thin-tailed breed). Also, we performed the association between copy number variation regions with fat-tailed deposition trait in these breeds and run subsequent bioinformatics approaches to report genes related to this trait.

## Methods

### Genotyping data

A total of 192 individual’s genomic data from 3 breeds including 96 Baluchi sheep^41^ and 47 Lori-Bakhtiari sheep^7^ as fat-tailed breeds and 47 Zel sheep^7^ as thin-tailed breed were used for this study. All samples were genotyped using the Illumina OvineSNP50 BeadChip array with 54,241 SNPs.

### SNP quality control

To obtain reliable and precise results for CNV detection, quality control (QC) was done in two phase including SNP genotyping and CNV calling. At SNP genotyping phase, we performed QC using Plink v1.07 software^42^. SNPs or samples were excluded if any of the following criteria was met: MAF < 0.01, (ii) animal call rate < 0.99, SNPs call rate < 0.95 and *p*-value for Hardy–Weinberg equilibrium < 0.00001. Also, the X and Y chromosomes were excluded from further analyses.

### CNV calling

The CNVs calling was implemented with the SNP data file from the GenomeStudio 1.0 software. The intensity files containing SNP name, chromosome, position, BAF and LRR were obtained for each dataset. The CNV analysis was performed by PennCNV v1.0.5 software^43^. This software uses different kind of data based on a hidden Markov model for CNV detection. First, the intensity files were converted into individual files using ‘-split’ option in PennCNV package. The individual-calling algorithm was performed using the ‘-test’ option. PennCNV includes GCmodel argument which utilizes a regression model for adjusting the high GC content and recovers samples influenced by genomic waves^44^. To adjust genomic waves, the ‘-gcmodel’ option with the ‘gcmodel’ file was implemented and GC content of the 1-Mb genomic region surrounding each marker (500 kb on each side) was measured. The additional input file for PennCNV including PFB (Population Frequency of B allele) was calculated based on the average BAF of each marker in the three breeds separately. If a large fraction of samples has waviness factor (WF value) less than -0.04 or higher than 0.04, it is much better to apply the adjustment procedure to reduce false positive calls (http://penncnv.openbioinformatics.org/en/latest/user-guide/test/). The status of CNV was classified into two classes: “loss” (CNV containing a deletion) and “gain” (CNV containing a duplication).

### CNVs quality control

The CNV filter was performed to enhance the accuracy of detected CNVs based on these criteria: (1) the CNV must contain at least ten SNPs without gap; (2) the length of the CNV more than 10 kb; and. Quality control was implemented in following rules: standard deviation of LRR < 0.3, BAF drift < 0.01 and a waviness factor between 0.05 and -0.05.

### Identifying CNVR

After CNV detection, the copy number variation regions (CNVRs) were identified by overlapping CNVs using the CNVRuler V1.2 program^45^. First, individual CNVs were merged into CNVRs, which are genomic regions covering CNVs overlapping by at least 1 bp^16^. This step is easy and straight, however, when the overlapping CNVs are highly long it can lead to overestimation of the size of CNVRs. To alleviate this issue, the CNVRuler gives the opportunity to evaluate base-by-base the regional density of the contributing CNVs and remove the low-density regions. Genomic regions with density lower than 10% were removed (“recurrence 0.1”)^45^. The recurrence trims a CNVR on the base of its occurrence to prevent false positive results, and it provides more reliable bounds of the regions^45^. The option "Gain/Loss separated regions" was used to assess the result (gain, loss) in each region. Overlapping "gain" and "loss" CNVRs were merged into single regions to identify genomic regions in which both gain and loss events can be observed ("mixed" CNVRs).

### Association analysis

To identify CNVRs significantly associated with fat deposition, a case–control analysis was performed using Fisher’s exact test on which fat-tailed sheep were treated as cases while thin-tailed sheep as controls. The genome wide association analysis between the CNVRs and the tail type was executed using CNVRuler V1.2 program^45^ and applying a logistic regression model. Bonferroni correction was used for solving the multiple testing problem. To balance the number of control samples against the cases and also to help focus the differences between the case and control groups on fat deposition, 70 samples of Valle del Belice breed^36^ were added to the association test as thin-tailed sheep breed. For Valle del Belice breed, quality control and CNV detection and filtration were performed exactly as previously mentioned for the other three breeds, and then the results were used for association analysis.

### Gene content in CNVRs

The BioMart database (http://www.biomart.org/) in Ensembl was used to investigate genes within or overlapping with the identified CNVRs for each sheep breed based on the Ovis aries (Oar_v3.1) gene sequence assembly. CNVRs overlapping with the coding region of the gene by at least 1 bp were considered to measure the proportion of CNVR overlapping genes^13^. Gene ontology and KEGG pathway analyses was performed in DAVID (http://david.abcc.ncifcrf.gov/). Because the genome annotation for sheep is not complete, we converted the ovine Ensembl gene IDs into human Ensemble gene IDs for the functional enrichment analysis.

### qPCR validation of CNVRs

To validate the detected CNVRs, qPCR analysis was used using five CNVRs that were selected randomly based on the results of PennCNV analysis. The primers (Additional file: Table S6) were designed using Primer 3 (http://bioinfo.ut.ee/primer3-0.4.0/) based on NCBI reference sequences. The genomic DNA of the same individuals in genotyping process was used for the experimental validation. For reference samples, four individuals with normal copy number were considered. The *DGAT1* gene was used as reference since it has been shown to have no variation in copy number in sheep genome^33^. PCR phases were implemented using Power SYBR Green PCR Reagent Kit (Applied Biosystems). The qPCR conditions with three replications for each sample were as: 95 °C for 3 min, followed by 40 cycles of 95 °C for 15 s and 60 °C for 60 s. We follow a standard 2^−ΔΔCt^ method to determine fold changes^46^ for which, to obtain the ΔΔC_t_, the ΔC_t_ value of a reference sample was compared with the sample of interest. To do a well comparison of copy number in all qPCR plots fold changes were normalized to a diploid number^13^. Finally, the copy number of the target regions was obtained through 2 × 2^−ΔΔCt^ equation. According to what described by Jiang et al., values of 2, 3 or more, and 1 or below approves normal, gain and loss events, respectively^19^.

## Results

### Quality control, CNVs and CNVRs

Table 1 shows the results of the raw data quality control. With strict quality control, most potentially problematic, low-quality data that reduce the reliability of CNVs are excluded from further analysis.

**Table 1:** Results of data quality control.

Removal criteria	Zel	Lori-Bakhtiari	Baluchi
Total animals	47	47	96
animal call rate < 0.99	4	3	9
Remaining animals	43	45	87
Number of SNPs	53,903	53,903	51,135
MAF < 0.01	1648	1945	2760
SNPs call rate < 0.95	3718	3884	224
HWE < 0.00001	69	98	29
unknown SNPs	31	30	25
Remaining SNPs	48,437	47,946	48,097

The CNVs calling were performed based on Illumina Ovine SNP50 Beadchip and PennCNV software. Table 2 represents the total number of CNVs detected by PennCNV in the Iranian sheep breeds.

**Table 2:** Copy number variation characteristics in two tail type sheep breeds.

Measurement	Fat-tailed			Thin-tailed		
	Gain	Loss	Total	Gain	Loss	Total
CNV count	273	300	573	164	78	242
Total length (Mb)	34.71	44.28	79.001	20.26	14.004	34.26
Average length(kb)	126.7	147.6	137.63	124.3	179.56	142.17
Median length(kb)	112.96	112.92	112.97	122.25	120.76	120.76
Max length(kb)	414	1729.93	1729.93	705.53	666.69	705.53
Min length(kb)	11.97	25.710	11.97	19.7	34.44	19.7
Average per sample(n)	2.3	2.52	4.82	3.8	1.81	5.61

**Figure 2:**
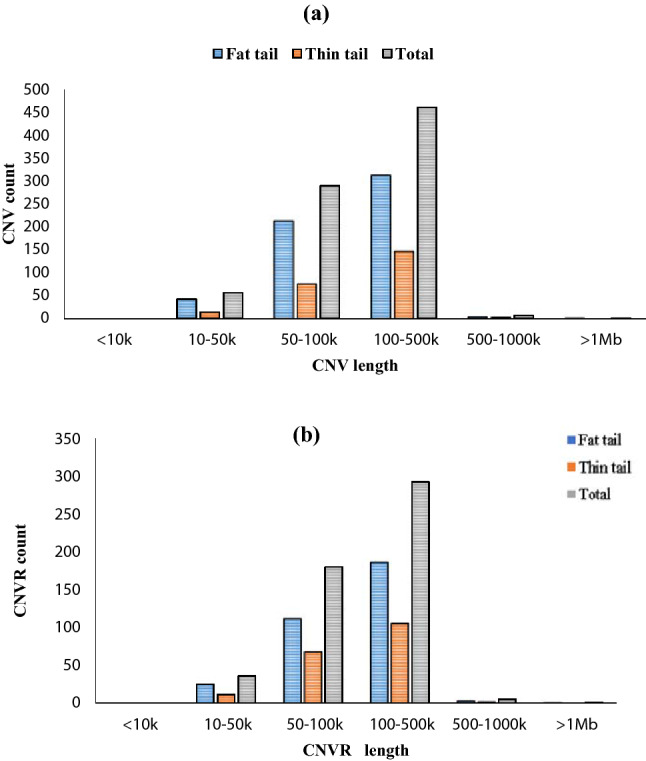
The distribution of CNVs size (**a**) and CNVRs size (**b**) in in fat and thin tail groups.

According to the PennCNV results, on 26 autosomal chromosomes a total of 815 CNVs including 573 and 242 CNVs were identified in fat tailed and thin tail sheep breeds, respectively. The CNVs length ranged from 11.97 kb (gain) to 1729.93 kb (loss) with an average of 137.63 kb and median size 112.97 kb in fat tailed, and from 19.7 kb (gain) to 705.53 kb (gain) with an average length of 142.17 kb and a median size of 120.76 kb in thin tail. The frequency of CNVs for each animal varied from 0.36% to 10.47% and the average number of CNVs in each individual ranged from 4.82 to 5.6 for fat and thin tail breeds, respectively. Our findings showed that in fat tail sheep, loss events were higher in number than gain events, whereas in thin tail sheep, gain events were higher in number than loss events. The results of the analysis showed that CNVs with a length range between 100 and 500 kb constituted 54.53% and 64.91% of the total CNVs in fat tail and thin tail sheep, respectively. CNVs shorter than 10 kb were not identified while CNVs longer than 1 Mb were detected with a frequency of 0.17 in fat-tailed (Fig. 2).

**Figure 3:**
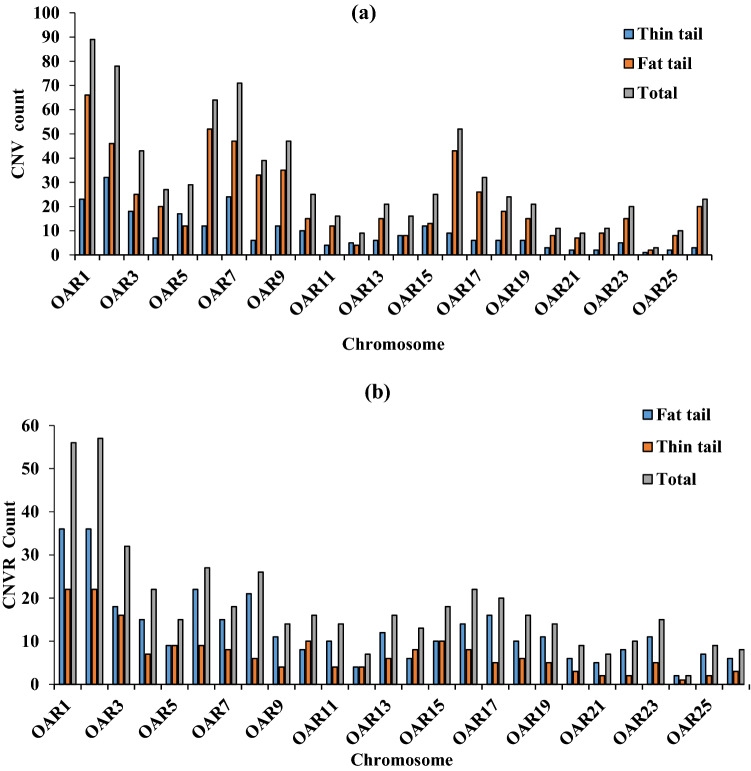
Distribution of the CNVs (**a**) and CNVRs (**b**) number on the 26 autosomal sheep chromosomes.

Results of CNV distribution in the 26 autosome chromosomes revealed the highest number of CNVs for fat tail on chromosomes 1, 6 and 2 with frequencies of 12.18, 9.27 and 8.36%, respectively. In thin tail, the highest number of CNVs was detected on chromosomes 2, 1 and 7 with frequencies of 13.22, 9.5 and 9.5%, respectively. In total, chromosomes 1, 2 and 7 had the most CNVs (Fig. S1). The maximum and minimum CNV length sizes were identified on Chromosome 10 (loss event) and on chromosome 1 (gain event) respectively. We found the number of CNVs in each chromosome to vary from 3 (chromosome 24) to 89 (chromosome1) and the average number of CNVs per chromosome was 30.42 (Fig. 3).

After merging overlapping CNVs, a total of 328 CNVRs (gain: 212, loss: 107, both: 9) and 187 CNVRs (gain: 152, loss: 34, both: 1) with lengths of 50.63 Mb, and 25.15 Mb were detected in fat tail and thin tail sheep breeds, respectively. Table 3 showed CNVRs information in two tail type Iranian sheep breeds. In total after merging overlapping CNVs in all samples, 483 CNVRs with a length of 73.85 Mb and an average of 146.89 kb were identified representing 2.6% of the entire sheep genome. Out of 483 CNVRs, 343 were gain, 126 were loss, and 14 were mixed within the same region. Most CNVRs sizes were identified in the range of 100 to 500 kb (57.74%). Also, shorter CNVRs lower than 10 kb were not observed. Also, chromosomes 1, 2, 3 and 6 had the highest CNVR and chromosomes 12, 24 and 21 had the lowest CNVR (Table S1).

**Table 3:** CNVRs Information in two tail type Iranian sheep breeds.

Measurement	Total	Loss	Gain	Mixed
Count	483	126	343	14
Length (Mb)	73.85	46.39	48.3	5.03
Average length (kb)	146.89	151.7	135.64	418.98
**Length range**				
< 10 kb	0	0	0	0
10- 50 kb	34(16.7%)	8(1.68%)	26(5.47%)	
50-100 kb	163(34.32%)	38(8%)	123(25.9%)	2(0.42%)
100-500 kb	273(57.74%)	74(15.58%)	191(40.21%)	8(1.68%)
500-1000 kb	4(0.84%)	1(0.21%)	2(0.42%)	1(0.21%)
> 1 Mb	1(0.21%)	0	0	1(0.21%)

**Figure 4:**
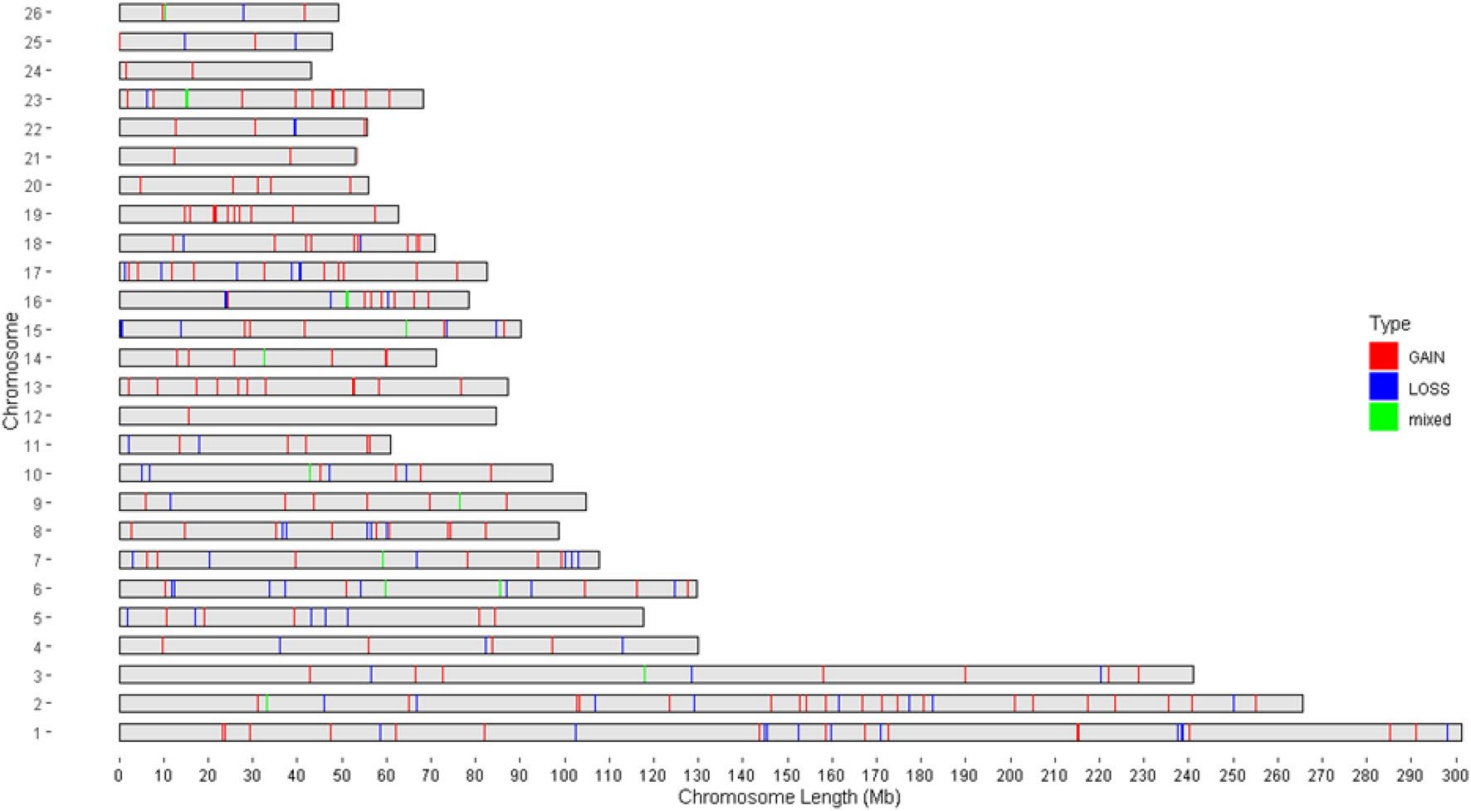
Genomic distribution and status of detected CNVRs in Iranian sheep breeds with two different types of tails.

Figure 4 shows the distribution of CNVRs on all chromosomes. As mentioned in material and methods chromosomes X and Y were excluded, respectively for poor coverage and to avoid gender effect. The map showed that CNVRs were not uniformly distributed across chromosomes and varied based on the position on each chromosome. The results showed that the number of CNVRs on chromosomes ranged from 2 to 57. Most CNVRs were located on larger chromosomes 2(57), 1(56), 3(32) and the lowest CNVRs were located on chromosomes 24(2), 12(2), 21(7). Also, the shortest and furthest distances between CNVRs were 26.351 kb and 29.38 kb, respectively on chromosome 13 and chromosome 5. In total, the average distance was 4.75 Mb between adjacent CNVRs.

The chromosomal regions covered by CNVRs (the total length of CNVRs detected per chromosome over chromosome length) varied between chromosomes, ranging from 0.91% in OAR12 to 4.34% in OAR16. The number of CNVRs on each chromosome varied from 2 on OAR24 to 57 on OAR2. Results showed that the number of CNVRs were positively related to chromosome length and this relationship was also linear (Table S1; Fig. S2).

### Association analysis

In comparative analysis between cases (two fat-tailed breeds) and controls (two thin-tailed breeds), one genomic region on chromosomes 6 was found to be in significant association with fat tail deposition (Table 4). The detected significant copy gained CNVR contained two significant protein-coding genes *HGFAC* and *LRPAP1*.

**Table 4:** association test between cases (fat tail sheep breeds) and controls (thin tail sheep breed).

CNVR	Chr	Start	End	Size	Freq (thin-tailed sheep)	Freq (fat-tailed sheep)	Type	Fisher’s exact test (P-value)	Gene(s)
1	6	114,604,408	114,720,361	115,953	11	0	Gain	0.00001	*HGFAC,LRPAP1*

### CNVRs gene content

Out of 483 CNVRs, 315 identified CNVRs (65.35%) overlapped with 790 genes, while remaining 167 CNVRs (34.64%) were in non-annotated gene regions. Further investigation showed that 77.72% of identified genes were of encoding protein, 9% of lincRNAs, 3.67% of miRNAs, 3.67% of snRNAs and 6% were other pseudogenes, processed pseudomorphs, snoRNAs, rRNAs, snRNA and misc_RNA. There were also 9 common genes between fat and thin tail sheep breeds (Table S2).

GO analysis showed that the annotation of these genes was involved in biological processes, cellular components, and molecular functions (Tables S3, S4). In the case of biological processes, genes were enriched in several terms such as adaptive immune response, cellular protein modification process, regulation of reactive oxygen species in thin-tailed breed and cellular protein modification process, regulation of heart rate, intestinal absorption in fat-tailed breeds. In molecular function annotation, these candidate genes were associated with protein-L-isoaspartate (D-aspartate) O-methyltransferase activity and phosphatidylinositol 3-kinase activity in thin tailed breed and Olfactory receptor activity, transcription factor binding and interleukin-10 receptor activity carbon monoxide binding, ATP binding in fat-tailed breeds. For the cell component annotation, genes were enriched in nuclear chromatin in thin-tailed breed and cytoplasm, mitochondrial respiratory chain complex in fat-tailed breeds.

According to the results of KEGG pathway analysis, these candidate genes were enriched in several signalling pathways, such as Regulation of lipolysis in adipocytes, Ras signalling pathway, Biosynthesis of antibiotics, Insulin resistance, Pantothenate and CoA biosynthesis, TNF signalling pathway, Melanoma, Glioma, Asthma and Regulation of lipolysis in adipocytes were significantly enriched (*p* < 0.05) (Table S5).

### CNVR validation by qPCR

In order to validate the obtained CNVRs by qPCR experiment, we randomly select five CNVRs with different types of CNV format (gain, loss). Four selected CNVRs were confirmed and were completely in accordance with PennCNV results (Table S6).

To evaluate the validity of the CNVRs identified in this study, the results were compared with 10 previous studies with different breeds, size, population structure, platform, and CNV identification algorithms (Table 5).

**Table 5:** Comparison of our study with recent ovine CNVR reports.

References	Assembly	Algorithm(s)	Platform	Sample size	Length range(kb)	CNVR count	Average length (kb)	Genome coverage (%)	Overlapping with this study (%)
This study	OaiAri3.1	PennCNV	OvineSNP50K	175	11.7–705.6	483	146.89	2.6	–
Fontanesi et al. (2011)	Btau_v4.0		Bovine aCGH385k	11	5.05–4.6	135	77.6		0.62
Liu et al. (2013)	OaiAri1	PennCNV	OvineSNP50K	327	13.66–300	238	253.57	2.3	10.16
Ma et al. (2015)	Oar_v3.1	PennCNV	OvineSNP50K	160	–	111	123.84	4.8	39
Hou et al. (2015)	OaiAri1	–	4.1 M aCGH	5	52–2000	51	304.86	0.6	0.62
Zhu et al. (2016)	Oar_v3.1	PennCNV	OvineSNP600K	110	–	490	225	3.3	7.05
Jenkins et al. (2016)	UMD3_OA	-	2.1 M aCGH	36	1–3600	3488	19	2.7	12.65
Ma et al. (2017)	Oar_v3.1	PennCNV	OvineSNP600K	48	1–2300	1296	93	4.7	28.63
Yan, et al. (2017)	Oar_v3.1	SVS	OvineSNP50K	385	15.3–6600	749	189	5.8	6.84
		PennCNV			11.4–2108.8	464	305.5		
		CnvPartion			87–12,093.7	104	1521.3		
Di Gerlando et al. (2019)	Oar_v3.1	SVS*	OvineSNP50K	468	16.62–1817	365	324.27	4.8	5.4

*Golden Helix SNP and Variation Suite (SVS).

## Discussion

The results of the present study showed that the frequency of the pattern of CNVs was different between the two groups of thin and fat-tailed sheep breeds in a way that fat-tailed breeds, loss events were higher in number than gain events, whereas in thin tailed breed, gain events were higher in number than loss events. Genetic and breed differences between the two groups could be a possible reason for this difference. Also, the status of CNVs may have been altered in adaptation to new environments and selective pressures. Iskow et al. studied the possible processes and evolutionary mechanisms that results in CNVs selection and reported that CNVRs facilitate the formation of novel genes in response to different environment adaptation^47^.

This may demonstrate that CNVs have been involved in the adaptation of fat tailed sheep to the harsh environment. Fat tail in sheep is an adaptive response to harsh environmental conditions and is a source of energy in adverse environmental conditions such as periods of food shortage and severe cold^48^. Salehian-Dehkordi et al. identified various CNV-related genes associated with climatic adaptation and artificial selection in sheep^29^.

We identified a total of 328 CNVRs with length 50.63 Mb and 187 CNVRs with length of 25.15 Mb in fat tailed and thin tailed breeds, respectively. Di Gerlando et al. identified a total of 365 CNVRs in Italian Insular Sheep Breeds and reported that the length of the CNVRs varied among breeds from 2.4 Mb to 124.1 Mb^49^. Ma et al. detected 111 CNVRs with an average size of 123.78 Kb in Chinese sheep using the 50 K SNP array. Coelho Ladeira detected 216 CNVRs from 491 Santa Inês individuals using the the 50 K SNP array^13^. Different results across studies may be due to different algorithms used for each study, quality control procidures,numbers of individuals and breed chrachteristics^50^.

Average copy number per animal was comparable with those reported in Chinese sheep; 14.16 by Liu et al.^51^, 49 by Winchester et al.^52^ and 15.4 in Valle del Belice sheep by Di Gerlando et al^36^. Bae et al. reported that increase in sample size leads to more detection of CNVs^53^. CNVR genome coverage (2.6%) in our study was similar to those reported by Liu et al. (2.3%)^34^ and Jekins et al. (2.7%)^54^ but lower than reports by Ma et al. (4.8%)^13^, Yan et al. (5.8%)^37^, Di Gerlando et al. (4.8%)^36^ and Ma et al. (4.7%)^40^. In other studies, CNVRs coverage in the sheep genome has been reported in the range of 0.6% to 5.8% (Table 4). Low coverage has also been observed in CNV studies in other studies^51,55,56^.

Beside breed specific differences and sample size, these differences could be attributed to the overestimation of the CNV calling algorithm by PennCNV. As the current CNV detection methods based on SNP arrays tend to give the false positives showing low correlation between multiple calling algorithms^57^. Also, because SNP arrays are inaccurate in genomic regions containing CNVs thus they lead to loss of CNV calling^52^. Moreover, segmental duplications (SDs) are considered as one of the prevalent hotspots for CNV formation that are influenced by low SNP coverage due to array design problems and implementation^5^.

The proportion of chromosomes covered by CNVR was different between the chromosomes and varied from 0.91% in OAR12 to 4.34% in OAR16. Zhu et al. in Chinese indigenous sheep with different types of tails identified the most CNVR on chromosomes 3 and 2 with coverage percentages of 4.56 and 2.9, respectively, and the lowest CNVR on chromosome 26^30^. Moreover, no CNVR was reported on chromosomes 25 and 26 in Tibetan sheep^30^.Ma et al. reported that the CNVR chromosomal coverage ranged from 1.5% to 18.2% on OAR24 and OAR26, respectively, while the number of CNVRs on each chromosome ranged from 15 on OAR23 to 135 on OAR3 concluding that there was a positive and strong linear relationship between the chromosome length and number of CNVRs (R^2^ = 0.87), which is similar to our study (R^2^ = 0.7702)^40^. Bae et al. reported the number of CNVRs per chromosome varied from 76 on chromosome 27 to 185 on chromosome 19 with a weak dependency of CNVRs number on the chromosome length (R^2^ = 0.27), which is not in agreement to the results of our study^53^.

Association analysis showed one significant copy gained region on chromosomes 6 (*p* < 0.001) harbouring two significant protein-coding genes *HGFAC* and *LRPAP1* involved in fat metabolism. Significant SNPs on chromosomes 6 related to fat deposition in sheep tail have already been reported through genome-wide association analyses^31,48^. The *HGFAC* serine proteases catalyses conversion of pro-HGF to active HGF^58^. The *HGFAC*, expressed in human omental adipose tissue of obese individuals^59^ is the activator of Hepatocyte growth factor (HGF) that has important role in obesity and cardiovascular disease^59^. *LRPAP* was discovered as a subunit of Low-Density Lipoprotein^60^. *LRPAP1* gene encodes the Receptor Associated Protein that influences the circulating levels of cholesterol in mice^60^. Also, the *LRPAP1* gene encodes the LDL receptor-related protein associated with early onset myocardial infarction. It is believed that the *LRPAP1* gene has a pleiotropic effect on various metabolic processes such as cholesterol homeostasis^61^. A recent GWAS on lipid traits and meta-analysis has shown an association of *LRPAP1* variant with Total cholesterol and LDL-C levels^62^.

Moradi et al. through a genome-wide detection of selection sweep identified three genomic regions located on chromosomes 5, 7 and X associated with fat deposition in sheep^7^. Zhu et al. identified *PPARA*, *RXRA*, *KLF11*, *ADD1*, *FASN*, *PPP1CA*, *PDGFA* and *PEX6* in fat-tailed and fat-rumped sheep, involving in fat tail deposition^30^. Paththinige et al. through genome-wide selection signature analysis identified genes influencing fat deposition (*NPR2*, *HINT2*, *SPAG8*, and tail formation (*ALX4*, *HOXB13*, *BMP4*) in Ethiopian Indigenous Sheep^62^. Li et al. reported *PDGFD* as causative gene for fat deposition in the tails of sheep using whole-genome resequencing of wild and domestic sheep^63^. Luo et al. using a whole-genome sequencing data reported that *GLIS1* may show essential contribution in the mesodermal cell differentiation throughout fetal development regulating fat deposition in sheep tails^64^. Wei Zhang et al. through, RNA sequencing data studied the transcriptome profiles of fat deposition in tail of sheep and identified *ABCA1* and *SLC27A2* genes as closely associated with tail phenotype^65^. Moradi et al. reported *BMP2* and *VRTN* as the underlying genes related to fat-tail phenotype in sheep^7^.

In this study CNVRs overlapped with 790 genes. Zhu et al. reported that CNVRs harboured 3130 genes among which *PPARA*, *RXRA*, *KLF11*, *ADD1*, *FASN*, *PPP1CA*, *PDGFA*, and *PEX6* were found in association with fat deposition^30^. In the present study, gene ontology resulted in several Zinc-Finger family genes including *ZNF354C*, *ZNF536*, and *ZNF804* in fat tail sheep breed, while *ZNF277* was the only enriched gene in thin tail breed. *ZNFs* are involved in transcriptional regulation, ubiquitin mediated protein degradation, signal transduction, actin targeting, DNA repair, cell migration and many other processes^66^. Liu showed that *ZNF* family (*ZNF622* and *ZNF688*) genes, which are highly expressed in some sheep breeds, are involved in regulating divergent and biologically different traits^67^.

Some olfactory receptors (ORs: LOC101113787, LOC101122949, LOC101122694) were identified in our study in fat-tailed sheep. Olfactory receptors have been reported in previous report by^34^. Long et al. observed many olfactory receptor genes in pigs with different copy numbers^68^. They stated that these CNVs may have been altered in adaptation to human environments and the feeding of pigs^68^. Also, Hou et al. identified numerous olfactory receptors (OR) genes in their study in Chinese sheep^35^. Positive selective pressures may lead to increased olfactory capabilities and eventually help adaptation to new environments. Studies have evidenced that ORs have multiple functions in different tissues concerning various physiological connections other than odor recognition. Wu et al. using a microarray analysis showed that cellular energy and lipid metabolism and obesity were regulated in the liver and adipose tissue of mice through high expression of olfactory receptor (receptor544)^69^. Mutlo et al. uncovered the molecular mechanisms behind the olfactory control of fat metabolism suggesting a connection between olfactory perception specificity of each individual and response to the obesity^70^. In natural environment organisms continually feel and adjust to changes in environmental, and sensory mechanisms particularly olfactory perception and its association with fat metabolism are key to processing these environmental changes and regulating physiological responses appropriatly.

Gene ontological analysis showed that, in fat-tailed sheep, the identified genes were significantly associated with the oxygen transporter activity and regulation of heart rate. The genes involved in these processes were associated with an increase in the number of copies. Many studies have provided results that the amount of oxygen in the adipose tissue microenvironment can influence adipose tissue metabolism and inflammation, and white adipose tissue oxygenation may, therefore, be a main contributor in the pathophysiology of not operating properly of adipose tissue^71,72^. Also, studies have demonstrated that acute exposure to severe hypoxia grew basal lipolysis in 3T3‐L1 adipocytes^73,74^. In addition, prolonged exposure to severe hypoxia moderately raised the amount of basal lipolysis, whereas low physiological oxygen partial pressure exposure increased lipolysis more in 3T3‐L1 adipocytes^75^. Also, it could be concluded that these regions are particularly related to adaptation at altitudes where oxygen concentration is reduced. Zhu et al. found the metabolic pathways related to oxygen transfer and heart rate regulator as well as two genes (*ALKBH5* and *NARFL*) associated with altitude adaptation in thin tail sheep^30^. Hou et al. stated that positive CNV changes in *HBB* and *CYP2C* genes may have contribution in adaptation to high altitudes and low oxygen environments in sheep^35^. It has been reported that the first wild sheep breeds were thin tail from which fat tail were generated as a consequence of natural and human selection^30^. These results suggest that natural and artificial selection for fat deposition in tail may result in copy number variations. Previous studies have shown that CNVRs are associated with environmental responses^76,77^. *PPAR-α* gene related to lipid synthesis was only identified in Zel breed (thin tail). *PPAR-α* is mainly found in the liver and plays an important role in regulating nutrient metabolism, including fatty acid absorption and oxidation^78^.

In the present study, response to starvation was the significant molecular function for which genes enriched in thin tail sheep. Yu et al. studied the effects of starvation on lipid metabolism and in Yak and reported that fat storage mobilization during starvation provides energy in yak^79^. This mechanism can be essential for supporting a regular metabolism that enables sheep survival during starvation condition. The homoeostatic mechanisms including increased lipolysis of adipose tissue and muscle proteolysis that provide non-esterified fatty acids and amino acids for gluconeogenesis, oxidation and ketogenesis are two key metabolic and physiological response to starvation^80,81^. Go analysis identified glucagone (GCG), adrenomedullin (ADM) and LDL receptor related protein 11 (LRP11) genes enriched in response to starvation. GCG acts directly on adipose tissue, including increasing blood flow, stimulation of lipolysis, increasing glucose absorption and oxygen consumption in brown adipose tissue. In addition, the administration of GCG also acutely increases the body's oxygen content in animals and human^82^.

Gene ontology showed that for molecular function, genes were significantly associated with transcription factor binding. Different transcription factors have been reported to be targets for regulation of fatty acid. This regulation is conducted by directly binding fatty acid to the transcription factors or by secondary process where signalling pathways are regulated by fatty acids that manage the expression of transcription factors^83^. Fatehi-Hassanabad et al. reported that the transcription factors such as PPARα, PPARγ and SREBP-1c responded to change in fat concentrations in tissues and accordingly regulate the genetic response to adopted metabolic conditions to assist either fat storage or catabolism^84^.

## Conclusions

A total of 328 and 187 CNVRs were detected in fat-tailed and thin-tailed sheep breeds, respectively. GO and KEGG pathway enrichment analysis demonstrated that these candidate genes were significantly involved in multiple signalling pathways. Also, 790 overlapping genes were found in this study. We identified one significant copy gained region on chromosome 6 containing *HGFAC* and *LRPAP1* genes that are involved in fat metabolism. We also showed that the diversity of the CNVs covers a significant portion of the sheep genome. However, due to the relatively small sample in our study, it is likely that a significant proportion of related CNVs to important gene regions are still unknown.

## Supplementary Information

Supplementary Information.

## Data Availability

The full dataset of Valle del Belice sheep are available from: https://www.animalgenome.org/repository/pub/UPIT2018.0803/. The data of Valle del Belice that support the findings of this study and the data of Iranian sheep breeds (Lori-Bakhtiari, Zel and Baluchi) are however available from the corresponding authors upon reasonable request.
