# Supplementary material for: Genome-wide identification of copy number variation and association with fat deposition in thin and fat-tailed sheep breeds
Source: Sci Rep. 2022 May 25;12:8834. doi: 10.1038/s41598-022-12778-1 (PMC9132911; doi:10.1038/s41598-022-12778-1)
Supplement: Supplementary file 1 — Supplementary Information. [file 41598_2022_12778_MOESM1_ESM.docx]

|  |
| --- |
| **Figure. S1. frequencies of CNVs on chromosomes in 2 tail types breed.** |

|  |
| --- |
| **Figure S2. Relationship between the number of CNVRs and chromosome length** |

| **Table S1. CNVR chromosomal distribution in Iranian sheep breeds** | | | | |
| --- | --- | --- | --- | --- |
| **Chromosome** | **CNVR count** | **CNVR length(mb)** | **Chromosome length^*^(mb)** | **Coverage (%)** |
| 1 | 56 | 6.57 | 301.31 | 2.18 |
| 2 | 57 | 6.82 | 265.69 | 2.57 |
| 3 | 32 | 3.88 | 241.14 | 1.61 |
| 4 | 22 | 2.05 | 130.07 | 1.58 |
| 5 | 15 | 2.48 | 117.63 | 2.11 |
| 6 | 27 | 3.63 | 129.79 | 2.79 |
| 7 | 18 | 3.32 | 107.7 | 3.08 |
| 8 | 26 | 3.52 | 98.77 | 3.57 |
| 9 | 14 | 2 | 104.71 | 1.92 |
| 10 | 16 | 1.87 | 97.21 | 1.92 |
| 11 | 14 | 1.84 | 60.68 | 3.02 |
| 12 | 7 | 0.77 | 84.52 | 0.91 |
| 13 | 16 | 2.6 | 97.26 | 2.97 |
| 14 | 13 | 2.001 | 71.11 | 2.81 |
| 15 | 18 | 2.87 | 90.32 | 3.17 |
| 16 | 22 | 3.4 | 78.35 | 4.34 |
| 17 | 20 | 3.39 | 82.58 | 4.09 |
| 18 | 16 | 2.31 | 70.86 | 3.26 |
| 19 | 14 | 2.66 | 62.71 | 4.24 |
| 20 | 9 | 1.19 | 55.94 | 2.13 |
| 21 | 7 | 0.97 | 52.95 | 1.82 |
| 22 | 10 | 1.79 | 55.67 | 3.21 |
| 23 | 15 | 2.62 | 58.31 | 3.84 |
| 24 | 2 | 0.47 | 43.08 | 1.13 |
| 25 | 9 | 1.3 | 47.66 | 2.74 |
| 26 | 8 | 1.11 | 49.39 | 2.24 |
| Total | 483 | 73.3 | 2869.51 | 2.6 |

| **Table S2. Gene component characterizes** | |
| --- | --- |
| Gene component | Frequency (%) |
| lincRNA | 8.99 |
| miRNA | 3.67 |
| misc_RNA | 1.01 |
| processed_pseudogene | 0.13 |
| protein_coding | 77.72 |
| pseudogene | 1.14 |
| rRNA | 1.14 |
| snoRNA | 2.53 |
| snRNA | 3.67 |

| **Table S3. The results of Gene Ontology in the fat tail** | | | |
| --- | --- | --- | --- |
| **Category** | **Term** | **Count** | ***P*-Value** |
| **Biological Process** |  |  |  |
| GO:0006464 | cellular protein modification process | 7 | 3.00E-03 |
| GO:0034394 | protein localization to cell surface | 4 | 3.10E-03 |
| GO:0045944 | positive regulation of transcription from RNA polymerase II promoter | 25 | 3.60E-03 |
| GO:0008630 | intrinsic apoptotic signaling pathway in response to DNA damage | 5 | 3.70E-03 |
| GO:0090557 | establishment of endothelial intestinal barrier | 3 | 9.20E-03 |
| GO:0002027 | regulation of heart rate | 4 | 9.90E-03 |
| GO:0050892 | intestinal absorption | 3 | 1.50E-02 |
| GO:0001953 | negative regulation of cell-matrix adhesion | 3 | 1.70E-02 |
| GO:0007215 | glutamate receptor signaling pathway | 3 | 1.70E-02 |
| GO:0016192 | vesicle-mediated transport | 7 | 1.70E-02 |
| GO:0034613 | cellular protein localization | 4 | 2.00E-02 |
| GO:0006974 | cellular response to DNA damage stimulus | 8 | 2.30E-02 |
| GO:0009083 | branched-chain amino acid catabolic process | 3 | 2.70E-02 |
| GO:0006535 | bundle of His cell to Purkinje myocyte communication | 2 | 2.70E-02 |
| GO:0086069 | cysteine biosynthetic process from serine | 2 | 2.70E-02 |
| GO:0055117 | regulation of cardiac muscle contraction | 3 | 3.20E-02 |
| GO:0010976 | positive regulation of neuron projection development | 5 | 3.30E-02 |
| GO:0019343 | atrioventricular valve formation | 2 | 4.00E-02 |
| GO:0003190 | cysteine biosynthetic process via cystathionine | 2 | 4.00E-02 |
| GO:0007050 | cell cycle arrest | 6 | 4.30E-02 |
| GO:0006468 | protein phosphorylation | 12 | 4.60E-02 |
| GO:0008652 | cellular amino acid biosynthetic process | 3 | 4.80E-02 |
| GO:0006259 | protein targeting to plasma membrane | 3 | 4.80E-02 |
| GO:0072661 | DNA metabolic process | 3 | 4.80E-02 |
| **Cellular Components** | **GO name** | **Count** | ***P* value** |
| GO:0005737 | cytoplasm | 89 | 7.80E-03 |
| GO:0005750 | mitochondrial respiratory chain complex III | 3 | 1.30E-02 |
| GO:0030054 | cell junction | 13 | 2.20E-02 |
| **Molecular Function** | **GO name** | **Count** | ***P* value** |
| GO:0008134 | transcription factor binding | 13 | 6.30E-04 |
| GO:0004920 | interleukin-10 receptor activity | 2 | 2.80E-02 |
| GO:0050421 | nitrite reductase (NO-forming) activity | 2 | 2.80E-02 |
| GO:0004124 | cysteine synthase activity | 2 | 2.80E-02 |
| GO:0070025 | carbon monoxide binding | 2 | 2.80E-02 |
| GO:0004984 | Olfactory receptor activity | 3 | 1.40E-02 |
| GO:0004122 | cystathionine beta-synthase activity | 2 | 2.80E-02 |
| GO:0004674 | protein serine/threonine kinase activity | 11 | 3.70E-02 |
| GO:0005524 | ATP binding | 30 | 4.10E-02 |
| GO:0070026 | nitric oxide binding | 2 | 4.10E-02 |

| **Table S4. The results of Gene Ontology in the thin tail** | | | |
| --- | --- | --- | --- |
| **Category** | **Term** | **Count** | ***P*-Value** |
| **Biological Process** |  |  |  |
| GO:0002250 | adaptive immune response | 10 | 2.80E-06 |
| GO:0006464 | cellular protein modification process | 8 | 1.50E-05 |
| GO:0051926 | negative regulation of calcium ion transport | 3 | 7.60E-05 |
| GO:0045671 | negative regulation of osteoclast differentiation | 3 | 6.90E-03 |
| GO:0006952 | defense response | 4 | 2.30E-02 |
| GO:1903426 | negative regulation of complement-dependent cytotoxicity | 2 | 2.80E-02 |
| GO:1903426 | regulation of reactive oxygen species biosynthetic process | 2 | 2.90E-02 |
| GO:0002767 | immune response-inhibiting cell surface receptor signaling pathway | 2 | 2.90E-02 |
| GO:0002774 | Fc receptor mediated inhibitory signaling pathway | 2 | 2.90E-02 |
| GO:0050776 | regulation of immune response | 6 | 2.90E-02 |
| GO:0030032 | lamellipodium assembly | 3 | 3.10E-02 |
| GO:1903660 | negative regulation of cell proliferation involved in contact inhibition | 2 | 3.70E-02 |
| GO:0072672 | neutrophil extravasation | 2 | 3.90E-02 |
| GO:0010155 | regulation of proton transport | 2 | 3.90E-02 |
| GO:0042594 | response to starvation | 3 | 3.90E-02 |
| GO:0019740 | nitrogen utilization | 2 | 4.60E-02 |
| **Cellular Components** | **GO name** | **Count** | ***P* value** |
| GO:0000790 | nuclear chromatin | 6 | 3.50E-02 |
| **Molecular Function** | **GO name** | **Count** | ***P* value** |
| GO:0004719 | protein-L-isoaspartate (D-aspartate) O-methyltransferase activity | 2 | 2.90E-02 |
| GO:0035004 | phosphatidylinositol 3-kinase activity | 2 | 4.70E-02 |

| **Table S5. KEGG pathways associated with CNVR genes** | | | |
| --- | --- | --- | --- |
| **Category** | **Term** | **Count** | ***P*-Value** |
| KEGG_PATHWAY | Rap1 signaling pathway | 12 | 3.30E-05 |
| KEGG_PATHWAY | Pantothenate and CoA biosynthesis | 4 | 5.40E-04 |
| KEGG_PATHWAY | Asthma | 5 | 7.80E-04 |
| KEGG_PATHWAY | Fc epsilon RI signaling pathway | 6 | 1.00E-03 |
| KEGG_PATHWAY | T cell receptor signaling pathway | 7 | 1.60E-03 |
| KEGG_PATHWAY | Insulin resistance | 7 | 2.60E-03 |
| KEGG_PATHWAY | Intestinal immune network for IgA production | 5 | 2.80E-03 |
| KEGG_PATHWAY | Fc gamma R-mediated phagocytosis | 6 | 2.90E-03 |
| KEGG_PATHWAY | mTOR signaling pathway | 5 | 3.00E-03 |
| KEGG_PATHWAY | ErbB signaling pathway | 6 | 3.20E-03 |
| KEGG_PATHWAY | Axon guidance | 7 | 4.00E-02 |
| KEGG_PATHWAY | Glioma | 5 | 4.30E-03 |
| KEGG_PATHWAY | Melanoma | 5 | 4.60E-03 |
| KEGG_PATHWAY | Measles | 7 | 5.70E-03 |
| KEGG_PATHWAY | Inflammatory bowel disease (IBD) | 5 | 6.80E-03 |
| KEGG_PATHWAY | TNF signaling pathway | 6 | 7.30E-03 |
| KEGG_PATHWAY | Biosynthesis of antibiotics | 9 | 7.90E-03 |
| KEGG_PATHWAY | Ras signaling pathway | 9 | 8.40E-03 |
| KEGG_PATHWAY | Regulation of lipolysis in adipocytes | 4 | 9.60E-03 |

| **Table S6. Designed Primers to CNVRs validation by qPCR.** | | | | | |
| --- | --- | --- | --- | --- | --- |
| CNVR no. | Position | Length(bp) | Type | Primers | validation |
| CNVR_357_1 | Chr16: 30432231:30493769 | 248 | loss | F-CACTGGAACATCACACACCC | yes |
|  |  |  |  | R-AAGAAGGGATGAGGGGAAGG |  |
| CNVR_375_1 | Chr17: 1808097:1862562 | 165 | loss | F- CCACCTGACTCCCCATTTCT | yes |
|  |  |  |  | R- TTACTCTGCCCCTGTCCTTG |  |
| CNVR_255_1 | Chr9: 7753367:7809711 | 233 | loss | F- AGGAGATGTGGGAGAGCAGA | yes |
|  |  |  |  | R- ACCTATGGAGACTACTTGCC |  |
| CNVR_424 | Chr20: 3665673:3753677 | 219 | gain | F- AGGCATTTGATTCTCCAGGC | no |
|  |  |  |  | R- TCCAGAGTGAGAGTAACGGG |  |
| CNVR_418 | Chr19: 44962949:44992524 | 221 | loss | F- AGTCCCATCGCAGTTTACCA | yes |
|  |  |  |  | R- AGGCTGTGAGGTGAAAAGGA |  |
| DGAT1 | Chr9 | 112 | normal | F-AGAATAGGGTCAGGGGCAGT |  |
|  |  |  |  | R- AGGACCCTAGGCCAGTCAAT |  |
